# Lateral shift of the femoral condyle after total knee arthroplasty: simulation using 2D-templates of the medial pivot design on knee radiographs of young Japanese patients

**DOI:** 10.1186/s13018-022-03342-8

**Published:** 2022-10-12

**Authors:** Arata Nakajima, Masato Sonobe, Yorikazu Akatsu, Manabu Yamada, Keiichiro Yamamoto, Junya Saito, Masaki Norimoto, Keita Koyama, Shinji Taniguchi, Hiroshi Takahashi, Yasuchika Aoki, Toru Suguro, Koichi Nakagawa

**Affiliations:** 1 Department of Orthopaedic Surgery, Toho University Sakura Medical Center, 564-1 Shimoshizu, Sakura, Chiba Japan; 2 Department of Orthopaedic Surgery, Faculty of Medicine, University of Tsukuba, 1-1-1 Tennodai, Tsukuba, Ibaraki 305-8575 Japan; 3 Department of Orthopaedic Surgery, Eastern Chiba Medical Center, 3-6-2 Okayamadai, Togane, Chiba 283-8686 Japan; 4 Department of General Medical Sciences, Graduate School of Medicine, Chiba University, 1-8-1 Inohana, Chuo-ku, Chiba 260-8677 Japan; 5 Japan Research Institute of Artificial Joint, 725-1 Sugo, Kisarazu, Chiba 292-0036 Japan

**Keywords:** Lateral shift, Femoral condyle, Total knee arthroplasty, Simulation, Medial pivot design

## Abstract

**Background:**

Total knee arthroplasty (TKA) is an established surgical treatment for advanced knee osteoarthritis by which patients can expect improvement of knee pain and function. Although many surgeons have investigated limb alignment after TKA, changes in coronal positional relation between the femur and tibia are not known well.

**Methods:**

Radiographs of 105 knees of young Japanese patients between 20 and 49 years-old (60 men and 45 women) without osteoarthritic changes who received arthroscopic surgeries at our hospital were used in this study. Using 2D-templates of the medial pivot design (the FINE total knee), we simulated TKA on a SYNAPSE-PACS software. First, the femoral component was placed in normal knee alignment and then was merged to the medial concave of the insert where the tibial component was placed in neutral alignment. The length of the mediolateral shift of the femoral component was measured as an estimate of lateral shift of the femoral condyle, of which association with radiographic parameters including the femorotibial angle (FTA), lateral distal femoral angle (LDFA), and medial proximal tibial angle (MPTA) was analyzed. Subjects were classified into three groups according to the femoral component size that was chosen in simulation of TKA, and the lateral shift of the femoral condyle was compared between groups.

**Results:**

The estimated mean lateral shift of the femoral condyle was 5.99 ± 1.98 mm and was greater in males than females (*p* < 0.05). Also, it was most highly correlated with the medial proximal tibial angle (MPTA) (*r* = − 0.553, *p* < 0.01). A group receiving larger component sizes significantly shifted more laterally compared with a group receiving smaller component sizes (*p* < 0.01).

**Conclusions:**

These results suggest that the coronal positional relation between the femur and tibia is altered and subsequent ligament imbalance may occur after mechanically aligned TKA using the medial pivot design.

## Background

The outcomes of total knee arthroplasty (TKA) are generally acceptable; however, approximately 20% of patients have some complaints after TKA [1–3]. The reasons for dissatisfaction after TKA remain poorly understood; however, one of the causative factors may be changes in coronal positional relation between the femur and tibia after TKA. In TKA using the medial pivot design, femoral components are restricted to the position of tibial inserts, which alters the normal positional relation between the femur and tibia. This may affect balance of major ligaments around the knee including medial collateral ligament (MCL), posterior cruciate ligament (PCL) (if the cruciate ligament-retaining type was used), lateral collateral ligament (LCL), and iliotibial tract (ITT).

The literature states that a neutrally aligned knee has a joint line that is oriented 3^o^ obliquely (alignment of 3^o^ varus in the tibia and 3^o^ valgus in the femur) [4, 5]. Nowadays, 22% of men and 17% of women among asymptomatic adult volunteers between 20 and 27 years-old have constitutional varus knees with a natural mechanical alignment of 3° varus or more [6]. Restoration of mechanical alignment to neutral in these cases may not be desirable and would be unnatural.

**Fig. 1:**
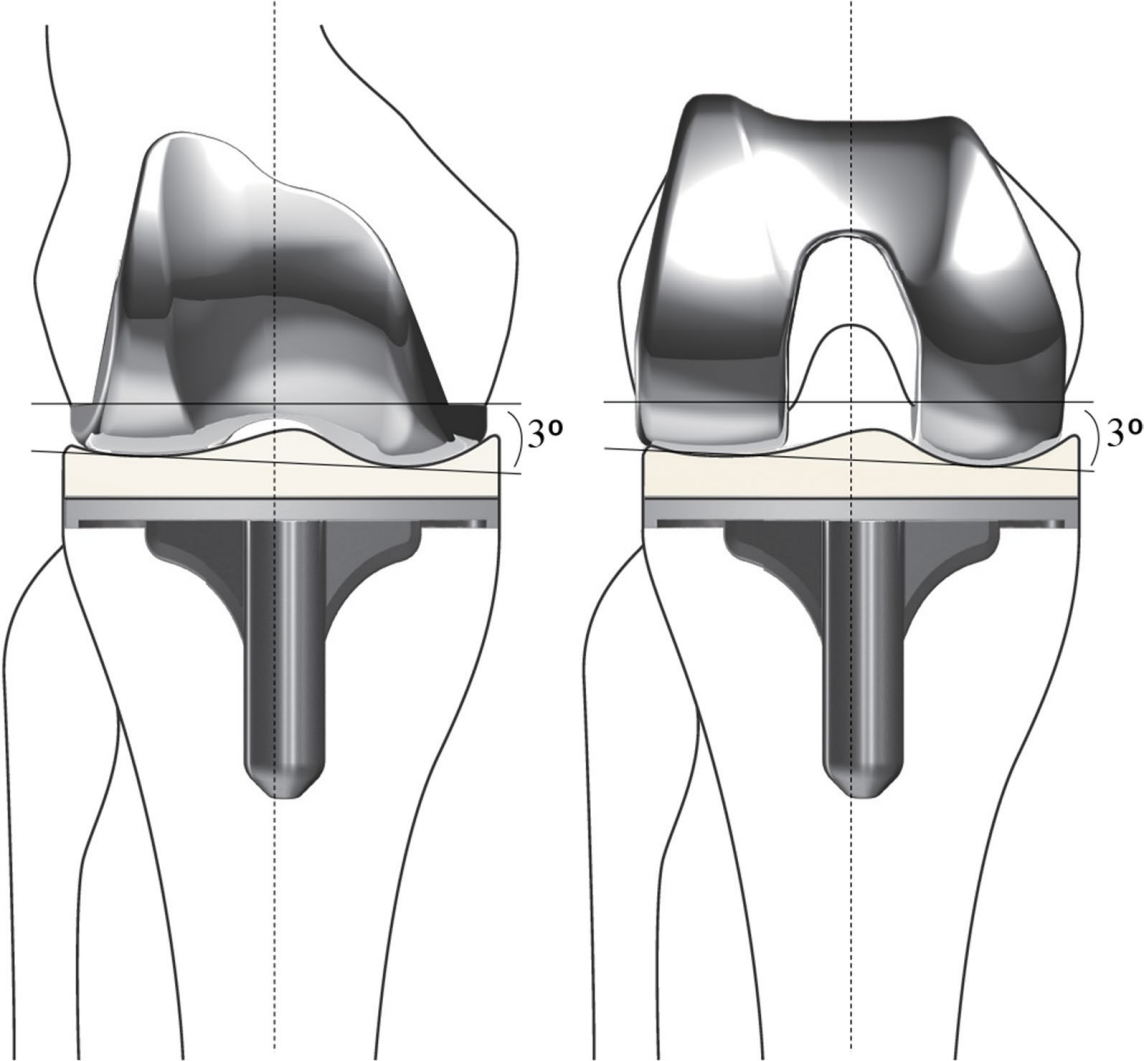
The FINE total knee. The femoral condyle has an asymmetric shape and femorotibial joint line with an oblique 3° both in coronal (left) and axial (right) planes which is incorporated into the implant design. The medial surface of the polyethylene insert has a convex curve while the lateral surface possesses a flat surface. FINE reproduces anatomical geometry by conducting osteotomy perpendicular to the mechanical axis. The figure is reprinted with minor modifications from Fig. 1 in the reference no. 7

The FINE total knee has characteristic design features including an oblique 3° femorotibial joint line. The design of the polyethylene insert includes a medial convex curve with increased conformity to the femoral component, and a lateral flat surface (Fig. 1). These features allow internal rotation of the tibia and femoral roll-back via medial pivot motion [7, 8]. However, there are concerns whether the increased medial conformity of the insert may alter the positional relation between the femur and tibia, which would affect the ligament balance.

Many investigators have paid attention to the coronal limb alignment of the hip-knee-ankle angle [1, 9–18]; however, to the best of our knowledge, there have been few reports looking at changes in coronal positional relation between the femur and tibia after TKA. In this study, using 2D-templates of the medial pivot design, we simulated TKA on knee radiographs of young Japanese patients without osteoarthritic changes. Our hypothesis was that the femoral condyle would be shifted laterally compared to its coronal position in normal alignment.

## Methods

### Patients

Radiographs of 105 knees without osteoarthritic changes were used. Patients were recruited from those between 20 and 49 years-old who received arthroscopic surgeries at our hospital between January 2018 and December 2020. There were 60 men and 45 women. Demographic data of the patients are shown in Table 1. The diseases included fifty-eight anterior cruciate ligament (ACL) injuries, thirteen medial meniscus tears, eleven lateral meniscus tears, seven plica synovialis, six PCL injuries, four cartilage injuries, two patella dislocations, two synovial tumors, one free body and one MCL injury.

**Table 1:** Demographic data of the patients and radiographic parameters of the knee

	Male (*n* = 60)	Female (*n* = 45)	*p*
Age (years)	35.8 ± 8.1	39.5 ± 7.9	0.021*
Height (cm)	173.2 ± 6.1	159.9 ± 5.2	0.000*
Weight (kg)	74.3 ± 12.0	60.6 ± 9.2	0.000*
BMI (kg/m^2^)	24.6 ± 3.4	23.7 ± 3.6	0.176*
FTA (^o^)	177.0 ± 1.9	176.0 ± 2.2	0.019*
LDFA (^o^)	81.2 ± 2.1	81.0 ± 2.2	0.679
MPTA (^o^)	84.8 ± 1.9	85.3 ± 1.7	0.161

BMI: body mass index, FTA: femorotibial angle, LDFA: lateral distal femoral angle, MPTA: medial proximal tibial angle. Significantly different. *p < 0.05

This study was approved by the institutional review board at our institution. All activities were performed in accordance with the ethical standards set forth in the Declaration of Helsinki, and informed consent was obtained from all patients who participated.

### Radiographic parameters, simulation of TKA using 2D-templates, and measurement of the lateral shift of the femoral condyle

The femorotibial angle (FTA), lateral distal femoral angle (LDFA), and medial proximal tibial angle (MPTA) were measured on the anteroposterior view of the knee radiographs according to our previous study [19].

**Fig. 2:**
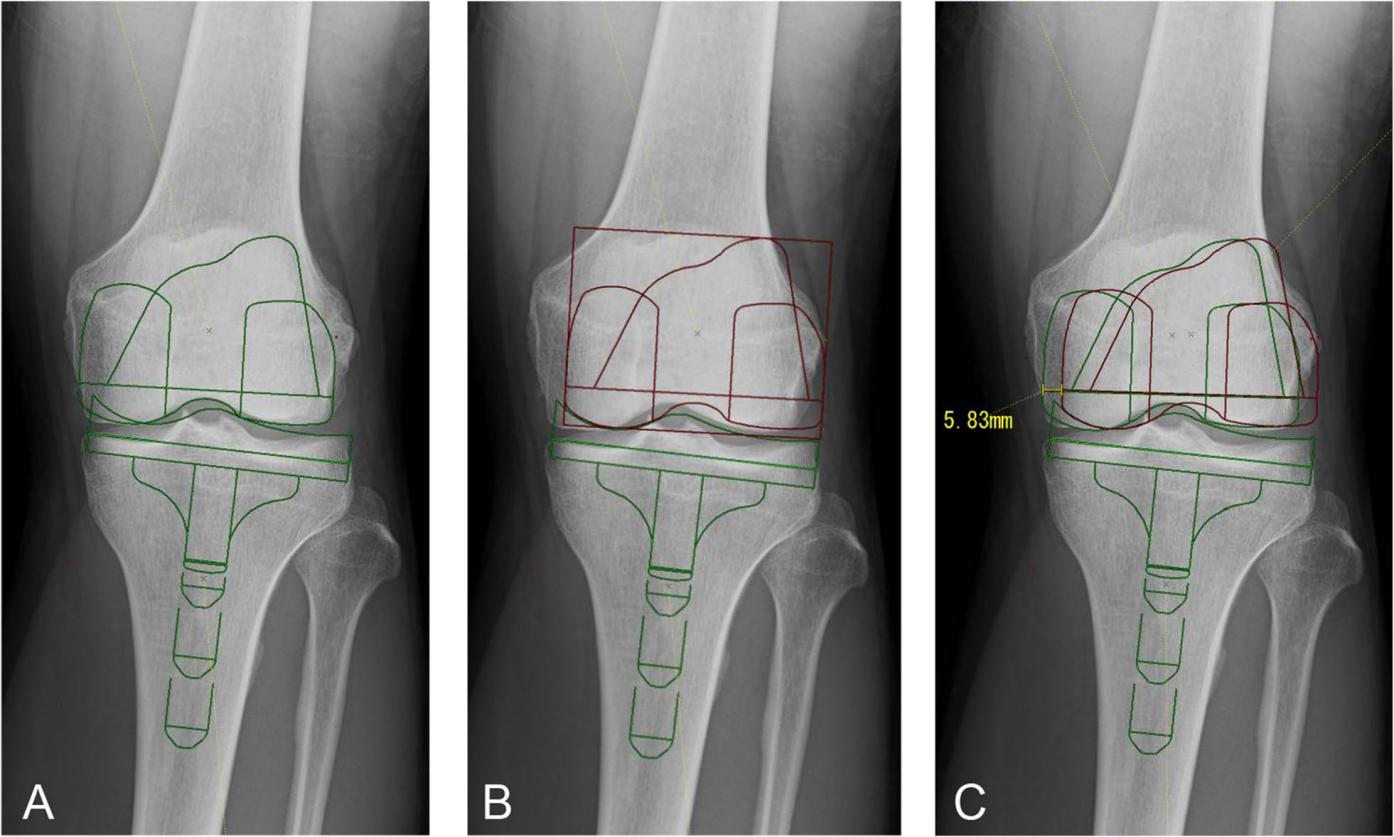
Simulation of TKA with the medial pivot design (the FINE total knee) on knee radiographs of young patients. The femoral component was placed to reproduce the form of the distal femoral condyles of the patients and not to overhang (**A**, green). The tibial component was placed in neutral alignment with the lateral joint line of the minimum thickness (7 mm) of the insert reproducing the original joint line. Then, we placed the femoral component to merge with the medial concave of the tibial insert (**B**, red). The length of the lateral shift of the femoral component was measured (**C**, a yellow bar). In this case, the 2L size was chosen and the lateral shift was 5.83 mm

We applied the 2D-templates of the FINE total knee (Teijin-Nakashima Medical, Okayama, Japan) to the anteroposterior view of the knee radiographs. First, the femoral component was placed to reproduce the form of the distal femoral condyles of each patient and not to overhang (Fig. 2A, green). The tibial component was placed in neutral alignment with the lateral joint line of the minimum thickness (7 mm) of the insert reproducing the original joint line. The size of the femoral and tibial components was chosen to be the same. Then we placed the femoral component so it merged with the medial concave of the tibial insert (Fig. 2B, red), which was supposed to be the postoperative alignment. The length of the mediolateral shift of the femoral component was measured and used as an estimate of the lateral shift of the femoral condyle (Fig. 2C, a yellow bar).

Three independent observers (AN, MS, YoA) measured the lateral shift of the femoral component. All measurements were performed using a SYNAPSE-PACS software (FUJIFILM, Tokyo, Japan).

### Classification for groups of the femoral component size

The FINE knee has eight size variations of the femoral component (S, S + , M, M + , L, L + , 2L, and 3L). Among them, 2D-templates for seven sizes except for L + were available in the software. The sizes were classified into three groups: group 1, S, S + and M; group 2, M + and L; group 3, 2L and 3L.

### Statistical analyses

The reliability of each radiographic measurement was assessed using inter- and intra-class correlation coefficients. All radiographic measurements in this study showed good reliability (all values > 0.8). Significance between groups was analyzed using the t-test or one-factor ANOVA. Results were expressed as the mean ± standard deviation (SD). Correlations between age, FTA, LDFA, MPTA, and the estimated lateral shift of the femoral condyle were statistically analyzed using the Pearson’s correlation coefficient. Data analyses were performed using SPSS software, version 21 (SPSS Inc., Chicago, IL, USA) and *p*-values < 0.05 were considered statistically significant.

## Results

### Radiographic parameters and comparison of estimated lateral shift of the femoral condyle between males and females

The mean FTA, LDFA, and MPTA for male and female subjects are shown in Table 1. The LDFA and MPTA were not statistically different between males and females, while the FTA was significantly greater in males than in females (*p* < 0.05). The estimated mean lateral shift of the femoral condyle for male and female subjects was 6.41 ± 1.95 mm and 5.43 ± 1.90 mm, respectively (Table 2). Males had a greater lateral shift of the femoral component than females (*p* < 0.05).

**Table 2:** Estimated lateral shift of the femoral condyle

	Male (*n* = 60)	Female (*n* = 45)	*p*
Lateral shift (mm)	6.41 ± 1.95	5.43 ± 1.90	0.011*

Significantly different. *p < 0.05

### Correlations between estimated lateral shift of the femoral condyle and the radiographic parameters

**Fig. 3:**
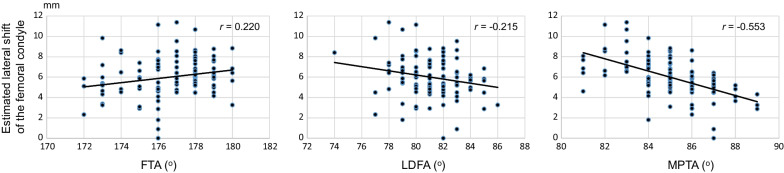
Correlations between the estimated lateral shift of the femoral condyle and radiographic parameters of the knee. The lateral shift of the femoral condyle was most highly correlated with MPTA (*r* = − 0.553), and weakly correlated with LDFA (*r* = − 0.215) and FTA (*r* = 0.220). MPTA, medial proximal tibial angle; LDFA, lateral distal femoral angle; FTA, femorotibial angle

Correlation coefficients among radiographic parameters are shown in Table 3. FTA was significantly correlated with LDFA (*r* = 0.505, *p* < 0.01) and MPTA (*r* = − 0.355, *p* < 0.01). There was a significant correlation between LDFA and MPTA (*r* = 0.309, *p* < 0.01). The lateral shift of the femoral condyle had the greatest correlation with MPTA (*r* = − 0.553, *p* < 0.01), and was weakly correlated with LDFA (*r* = − 0.215, *p* < 0.05) and FTA (*r* = 0.220, *p* < 0.05) (Fig. 3).

**Table 3:** Correlations between radiographic parameters and lateral shift of the femoral condyle

	Age	FTA	LDFA	MPTA	Lateral shift
Age		− 0.024	0.132	0.142	− 0.027
FTA			0.505**	− 0.355**	0.220*
LDFA				0.309**	− 0.215*
MPTA					− 0.553**

FTA: femorotibial angle, LDFA: lateral distal femoral angle, MPTA: medial proximal tibial angle. Significantly different. *p < 0.05, **p < 0.01

### Comparison of the lateral shift of the femoral condyle between its size groups

**Fig. 4:**
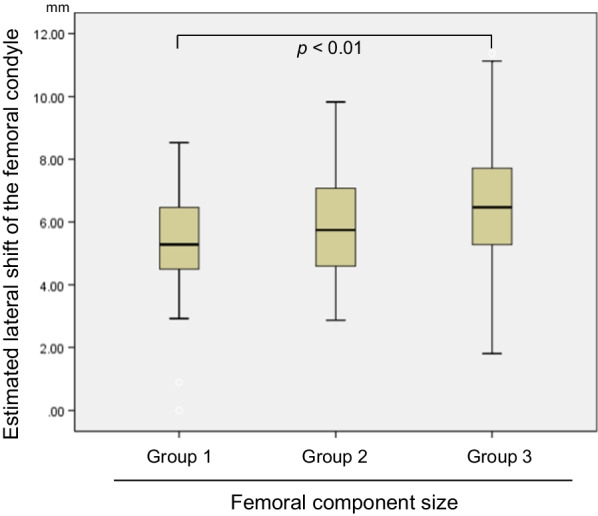
Comparison of the estimated lateral shift of the femoral condyle between size groups for the FINE knee. Group 1 includes sizes for S, S + and M; group 2 for M + and L; group 3 for 2L and 3L. Group 3 significantly shifted more laterally compared with group 1 (*p* < 0.01). Thick horizontal line: median value; box: interquartile range (IQR); whiskers: most extreme points within 1.5 times the IQR from the limits of the box

The estimated mean lateral shift for all cases was 5.99 ± 1.98 mm. The mean lateral shift was 5.24 ± 1.84 mm for group 1, 5.99 ± 1.78 mm for group 2, and 6.56 ± 2.02 mm for group 3 (Fig. 4). Group 3 significantly shifted more laterally compared with group 1 (*p* < 0.01), and the mean difference was 1.31 ± 0.42 mm. There was no significant difference in the lateral shift between groups 2 and 3. Group 1 and 3 included females and males alone, respectively. Fifty-nine percent (13/22) of group 2 were males.

## Discussion

In this study, we demonstrated that the femoral condyle was shifted laterally by an average of 5.99 mm relative to its position where the implant with the medial pivot design was tentatively placed in normal alignment. Interestingly, the lateral shift was significantly correlated with the MPTA (*r* = − 0.553). According to Bellemans et al., approximately 20% of young adult volunteers have constitutional varus knees with a natural mechanical alignment of 3° varus or more [6]. These observations suggest that the lateral shift of the femoral condyle will be greater when patients with constitutional varus alignment receive TKA with the medial pivot design. The lateral shift of the femoral condyle alters the coronal positional relation between the femur and tibia, which may affect balance of ligaments including MCL, PCL, LCL and ITT. Imbalance of those ligaments may cause inappropriate tension of ligaments during knee motion and lead to poor clinical outcomes.

As most patients receiving TKA, except for a bi-cruciate ligament-retaining type, lack ACL function, the tibial insert has a medial concave design to increase stability and allows medial pivot motion. However, this study shows that the medial concave of the insert restricts the position of the femoral component and shifts laterally after TKA. Those are concerns in TKA with the medial pivot design. Clinical studies comparing flat vs. concave inserts have been made and reported [20, 21]. Uvehammer et al. demonstrated that the Hospital for Special Surgery knee score and the patients’ opinion based on their preoperative expectations were not very different [20]. On the other hand, a two-year matched pair cohort study demonstrated that the clinical outcomes of the medial pivot design used in CR-TKA were more favorable than those of the flat surface design [21]. It is difficult to draw conclusions about which is better, but the absence of significant differences in clinical scores shown by Uvehammer et al. suggests that even the flat surface allows medial pivot motion when appropriate ligament balances are achieved.

The current study clearly showed that the lateral shift of the femoral condyle was associated with the MPTA in simulations of TKA from the young Japanese knee radiographs. To maintain the normal positional relation between the femur and tibia after TKA, it is important to consider pre-arthritic alignment of each patient [22–25]. To date, studies have reported anatomical variations between the femur and tibia in young adult knees [10–12, 14–18, 26]. In a report including 23 Japanese and 47 Caucasian healthy young subjects, Japanese subjects had a significantly greater varus alignment than Caucasians, while women exhibited a more valgus alignment than men [16]. Racial and gender differences in knee-joint obliquity also were reported by Tang et al. Compared with Caucasian subjects, Chinese subjects had significantly larger medial inclination of the knee joint (knee-joint obliquity) and female Chinese subjects had significantly more varus alignment than female Caucasians [18]. Furthermore, Hirschmann et al., using 3D-reconstructed CT scans, demonstrated that there was variability of knee phenotypes in young non-osteoarthritic knees. They showed that in males, the most frequent combination (knee phenotype) was a neutral phenotype in the femur and a neutral phenotype in the tibia, while in females, it was a neutral femoral phenotype and a valgus tibial phenotype [26]. Taken together, these observations suggest that there are racial and gender differences in normal knee alignment and a more individualized approach will be necessary to achieve better clinical outcomes after TKA.

In terms of coronal lower limb alignment in Japanese normal knees, Nakano et al. showed that the femoral condylar orientation and tibial plateau inclination in young and middle-aged (15–54 years-old) participants were greater in males than females [27]. For male participants in their study, the tibial plateau inclination was 85.1 ± 2.4° in the young and 85.6 ± 2.1° in the middle-aged populations. These angles seem to be smaller than the measurements for similar aged populations by Hirschmann et al. who showed that the tibial mechanical angle was 86.7 ± 2.3° for males and 88.0 ± 2.4° for females [26]. In the current study, the estimated lateral shift of the femoral condyle was greater in males than in females. Although there was no significant difference in the MPTA between males and females (Table 1), small component sizes (group 1) were used in females alone and large sizes (group 3) were used in males alone. This may affect differences in the lateral shift of the femoral condyle between males and females. Taking these findings into account, when planning TKA for Japanese patients, individual tibial plateau inclination should be considered to reconstruct pre-arthritic alignment especially for male patients.

In this study, we showed that the lateral shift of the femoral condyle in the coronal plane would occur after TKA using the medial pivot design. Although the data were not shown, the similar shift would occur in the axial plane as an asymmetric shape and femorotibial joint line with an oblique 3^o^ is also incorporated in the axial plane of the implant design (Fig. 1). This may also cause inappropriate tension of ligaments including MCL, PCL, LCL and ITT in flexion.

Mediolateral femoral component position also affects patellar shift and femoral roll-back. Studies using fresh cadaver specimens demonstrated that following mediolateral translation of the femoral component, the patella was significantly shifted and tilted in the same directions, while retropatellar pressure was not significantly altered [28]. More than eight years of follow-up of TKA by van de Groes et al. showed that a medialization of ≥ 5 mm resulted in a significantly lower anterior knee pain score [29]. These results imply that a lateral shift of the femoral condyle increases the risk of patellofemoral problems such as anterior knee pain in the long-term.

This study has some limitations. First, the sample size was relatively small, and the subjects included patients with meniscus (24 cases) or ligament (64 cases) injuries whose knee alignment may not have been completely normal. Second, the lateral shift of the femoral component was measured on radiographs using 2D-templates of only one knee prosthesis (the FINE total knee) and not compared with other prostheses. As the estimated lateral shift is affected by implant size variations, it will change when other prostheses are used in simulations. Similar studies using 3D-templates are desirable to better understand the positional relation between the femur and tibia. The present data clearly showed that a lateral shift of the femoral condyle would occur after mechanically aligned TKA with the medial pivot design, which may affect ligament balance; however, biomechanical analyses were not undertaken in this study. Future studies using computational simulation or cadaver specimens are required to evaluate ligament balance or knee kinematics.

## Conclusions

The femoral condyle was shifted laterally compared with its coronal position in normal alignment when simulating TKA with the medial pivot design, and the lateral shift was greater in males than in females. Further studies of the positional relation between the femur and tibia or ligament balance that may be affected by the lateral shift of the femoral condyle are required to better understand knee kinematics after TKA using the medial pivot design.

## Data Availability

The datasets used and/or analyzed during the current study are available from the corresponding author on reasonable request.

## Abbreviations

TKA: Total knee arthroplasty
MCL: Medial collateral ligament
LCL: Lateral collateral ligament
ACL: Anterior cruciate ligament
PCL: Posterior cruciate ligament
ITT: Iliotibial tract
LDFA: Lateral distal femoral angle
MPTA: Medial proximal tibial angle
FTA: Femorotibial angle
